# Identifying unmet clinical need in hypertrophic cardiomyopathy using national electronic health records

**DOI:** 10.1371/journal.pone.0191214

**Published:** 2018-01-11

**Authors:** Mar Pujades-Rodriguez, Oliver P. Guttmann, Arturo Gonzalez-Izquierdo, Bram Duyx, Constantinos O’Mahony, Perry Elliott, Harry Hemingway

**Affiliations:** 1 Leeds Institute of Biomedical and Clinical Sciences, University of Leeds, Leeds, United Kingdom; 2 Farr Institute of Health Informatics Research, Institute of Health Informatics, University College London, London, United Kingdom; 3 Institute for Cardiovascular Science, University College London Institute for Cardiovascular Science and Barts Heart Centre, St. Bartholomew’s Hospital, St Bartholomew’s Hospital, London, United Kingdom; 4 CAPHRI School for Public Health and Primary Care, Maastricht University, Universiteitssingel, Maastricht, the Netherlands; University of California, Davis, UNITED STATES

## Abstract

**Introduction:**

To evaluate unmet clinical need in unselected hypertrophic cardiomyopathy (HCM) patients to determine the risk of a wide range of subsequent cardiovascular disease endpoints and safety endpoints relevant for trial design.

**Methods:**

Population based cohort (CALIBER, linked primary care, hospital and mortality records in England, period 1997–2010), all people diagnosed with HCM were identified and matched by age, sex and general practice with ten randomly selected people without HCM. Random-effects Poisson models were used to assess the associations between HCM and cardiovascular diseases and bleeding.

**Results:**

Among 3,290,455 eligible people a diagnosis of hypertrophic cardiomyopathy was found in 4 per 10,000. Forty-one percent of the 1,160 individuals with hypertrophic cardiomyopathy were women and the median age was 57 years. The median follow-up was 4.0 years. Compared to general population controls, people with HCM had higher risk of ventricular arrhythmia (incidence rate ratio = 23.53, [95% confidence interval 12.67–43.72]), cardiac arrest or sudden cardiac death (6.33 [3.69–10.85]), heart failure (4.31, [3.30–5.62]), and atrial fibrillation (3.80 [3.04–4.75]). HCM was also associated with a higher incidence of myocardial infarction ([MI] 1.90 [1.27–2.84]) and coronary revascularisation (2.32 [1.46–3.69]).The absolute Kaplan-Meier risks at 3 years were 8.8% for the composite endpoint of cardiovascular death or heart failure, 8.4% for the composite of cardiovascular death, stroke or myocardial infarction, and 1.5% for major bleeding.

**Conclusions:**

Our study identified major unmet need in HCM and highlighted the importance of implementing improved cardiovascular prevention strategies to increase life-expectancy of the contemporary HCM population. They also show that national electronic health records provide an effective method for identifying outcomes and clinically relevant estimates of composite efficacy and safety endpoints essential for trial design in rare diseases.

## Introduction

With an estimated prevalence of 0.2% in young adults[1], hypertrophic cardiomyopathy (HCM) is the most common genetic disorder of the cardiac muscle. This genetic disorder is associated with premature death from sudden ventricular arrhythmia, heart failure and stroke[2,3].

Estimating the long-term health burden of rare diseases such as HCM through retrospective cohort studies and registries is difficult given the small number of people affected by the conditions. This gap in evidence is one reason for a failure to conduct randomised trials powered to detect clinically meaningful endpoints in HCM. In this study we hypothesised that population-based electronic health records (EHR) can be used to identify the extent of unmet clinical need in people with rare diseases such as HCM and thereby determine relevant outcome and safety endpoints for use in trials.

By using the ClinicAl research using LInked Bespoke studies and Electronic health Records (CALIBER) resource, which has been extensively validated in studies of cardiovascular risk factors[4–10], myocardial infarction (MI)[11], and heart failure (HF)[12] in England, we sought to develop an algorithm for the identification of cases of HCM and use it to provide data on clinically relevant endpoints and potential trial safety measures.

## Methods

### Study population

We evaluated 5.37 million people’s records from 225 general practices in England providing data to the CALIBER platform (Figure A in S1 File). CALIBER links individual EHR across four data sources–primary care from the Clinical Practice Research Datalink (CPRD)[13] coded with the Read system (which maps to the Systematized Nomenclacture of Medicine–Clinical Terms, SNOMED-CT[14]); hospital data from the Myocardial Ischaemia National Audit Project disease registry[15] and the Hospital Episodes Statistics; and the national death registry (both coded with the International Classification of Diseases, ICD-10). The primary care practices in CPRD are representative of the UK primary care setting[16,17] and patients are representative of the general population in terms of age, sex, ethnicity[17,18] and overall mortality[19]. Both cardiovascular endpoints and risk factors have been validated for epidemiological research [4,6–9].

### People with HCM

**Fig 1:**
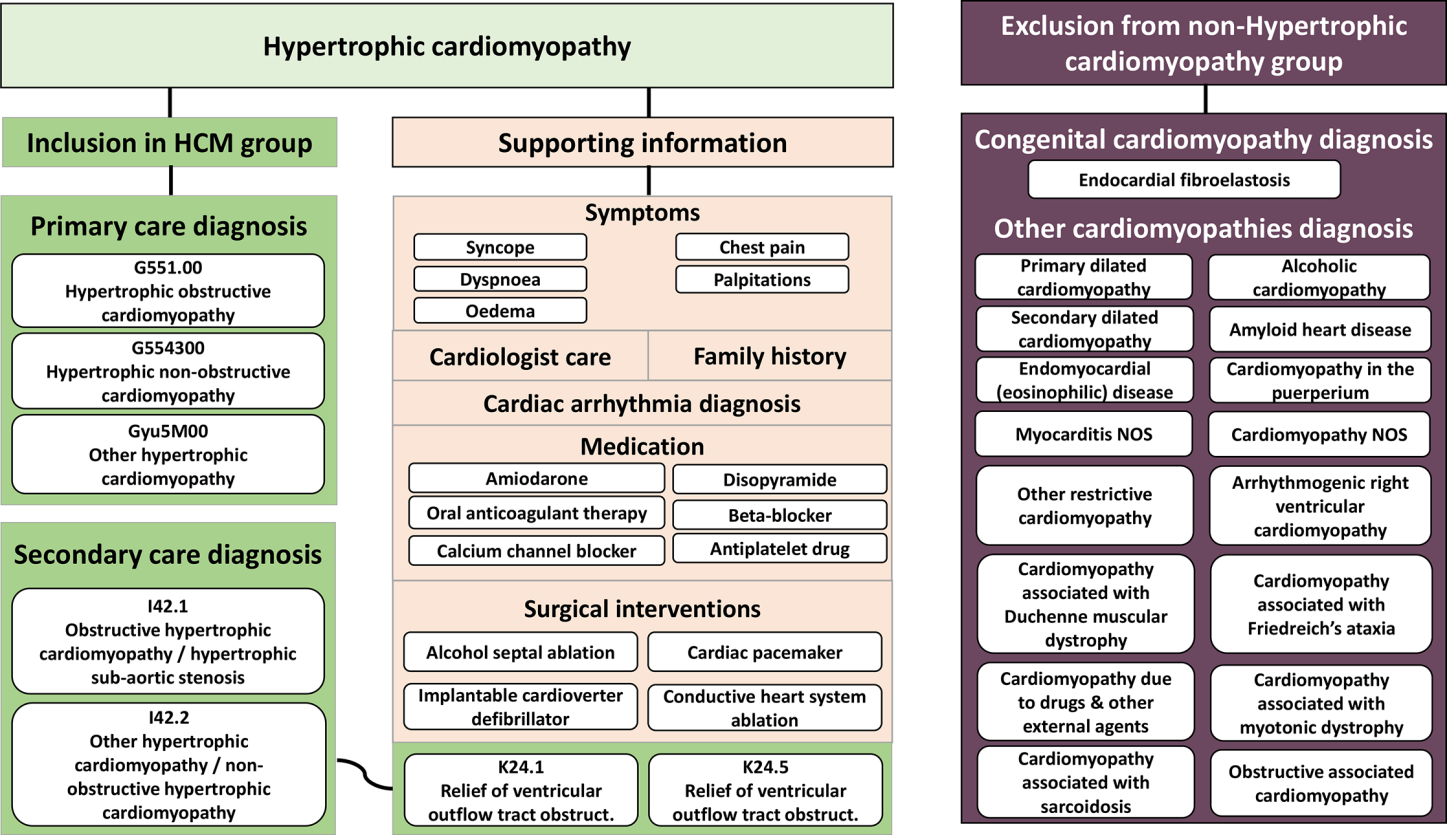
Defining cases of hypertrophic cardiomyopathy and non hypertrophic cardiomyopathy in national samples of structured electronic health records: phenotype algorithm using multiple ontologies (ICD-10, Read-2, OPCS-4, BNF). [Fig note: The phenotype algorithm uses multiple ontologies: the International Classification of Disease version 10 (ICD-10), the Read classification system, the Office of Population Censuses and Surveys Classification of Intervention and Procedures (OPCS) and the British National Formulary (BNF). Grey shaded areas indicate how the algorithm was constructed for the definition of hypertrophic cardiomyopathy (HCM) in primary analysis. Record of symptoms, cardiologist care, cardiac arrhythmia, medication and/or surgery within +/-6 months of a recorded HCM diagnosis was considered evidence of supporting information of HCM. HA, hospital admissions; PC, primary care.].

The approach to defining HCM in linked EHRs follows previously published methods for MI, HF, atrial fibrillation (AF), rheumatoid arthritis or polymyalgia rheumatica[5,11,12,20,21] All patients with HCM, registered in the general practices contributing data to CALIBER[22] between January 1997 and March 2010 were identified. Those who had less than one year of follow-up after practice registration were excluded. HCM was defined based on codes for diagnosis and invasive therapy for left ventricular outflow tract obstruction in EHR from primary care (Read codes) and hospital admissions (ICD-10 and OPCS codes; Fig 1). Additional information was extracted to conduct sensitivity analyses to indirectly assess the validity of our definition of HCM by examining the robustness of estimates after restricting the analyses to patients who had supporting information of HCM diagnosis. This information included: family history of HCM (n = 60), cardiac arrhythmia (n = 244); surgical interventions (n = 153), symptoms (n = 138) or medication (n = 390) recorded within 6 months of HCM diagnosis; a referral to a cardiologist in the 6 months before HCM diagnosis (n = 8); and capture of HCM diagnosis in both primary care and hospital (n = 331).

### General population controls without HCM

Each individual with HCM was matched to up to ten randomly selected people without HCM identified amongst those actively registered in the same general practice on his/her date of inclusion (index date). HCM and non-HCM individuals were matched for age (±5 years) and sex, and had a minimum of one year of follow-up after practice registration. People with diagnosis of other cardiomyopathies were excluded from the non-HCM comparison group. Prior to the start of the study, it was estimated that including 12,760 individuals (1160 patients with HCM and 10 randomly selected patients without HCM per exposed individual), and assuming 80% power, a 2-sided 5% significance level and 10% probability of a cardiovascular endpoint, would allow detecting a minimum effect size of 1.17 in a Cox Proportional Hazard analysis (Stata/MP 13.1).

### Risk factors, co-morbidities and treatments

Described patient characteristics recorded in primary care were: sex, age, index of multiple deprivation, ethnicity, personal and family history of cardiomyopathy, cardiovascular risk factors (smoking status, diagnosis of diabetes mellitus, hypertension, systolic blood pressure, body mass index, and serum lipids), and prescribed medication (blood pressure lowering medication, statins, anticoagulant and antiplatelet drugs). Baseline characteristics were defined as the most recent information recorded in primary care up to one year before study entry, a diagnosis recorded at any time before or on the date of entry, or the recording of ≥2 drug prescriptions in the previous year. Hypertension was defined as a diagnosis recorded at any time before or on the date of entry, or a minimum of 3 measurements of raised systolic or diastolic blood pressure in the year prior to the date of study entry. Raised systolic blood pressure was defined as ≥140 mmHg (≥130 mmHg for patients with diabetes). Raised diastolic blood pressure was defined as ≥90 mmHg (≥80 mmHg for patients with diabetes). Definitions can be found at https://www.caliberresearch.org/portal/.

### Endpoints

We evaluated four groups of fatal and non-fatal disease endpoints. First, disorders which are known to be strongly associated with HCM, and which constitute a validity check of the disease definition. These include cardiac arrest or sudden cardiac death (CA-SCD), ventricular arrhythmia, AF, stroke and HF. Second, individual cardiovascular diseases where the evidence that HCM is associated with an increased risk is less clear. These include coronary endpoints, peripheral arterial disease (PAD), abdominal aortic aneurysm and a composite of deep venous thrombosis and pulmonary embolism (DVT-PE). Third, we evaluated plausible composites that could be/have been the basis of primary endpoints in clinical trials, including: all-cause mortality, and cardiovascular mortality, MI and stroke. Fourth, we evaluated gastrointestinal and major bleeding as potential trial safety endpoints. Definition of major bleeding was a bleeding cause of death in the death registry or all cause death within 7 days of a bleeding record in primary care or hospital admission, a hospital admission for 14 days or more or a transfusion record in primary or hospital care within 30 days of a bleeding record. Sites of major bleeding considered were intracranial, gastrointestinal, respiratory and ocular. Diagnosis codes used to define each endpoint can be found in Table A in S1 File.

### Study design and follow-up period

All (100%) eligible individuals contributed follow-up information to the cohort study. For people with HCM follow-up began on the date of first recorded HCM diagnosis (or date of the invasive therapy for left ventricular outflow tract obstruction for HCM patients without recorded HCM diagnosis) or on the date on which the patient was eligible for inclusion, if the diagnosis was recorded earlier. For people without HCM the follow-up began on the index date of the matched HCM patient. For individuals with and without HCM, the follow-up ended on the first occurrence of the following: death, reaching a cardiovascular or bleeding endpoint, or leaving (de-registering) from their general practice.

### Statistical analysis

We used descriptive statistics (mean and standard deviation [SD] or median and interquartile range [IQR], as appropriate for continuous variables; and frequencies and percentages for categorical variables) to describe baseline individual characteristics and the frequency of any of the morbidity endpoints recorded before study entry (history of cardiovascular endpoints) in people with and without HCM.

**Fig 2:**
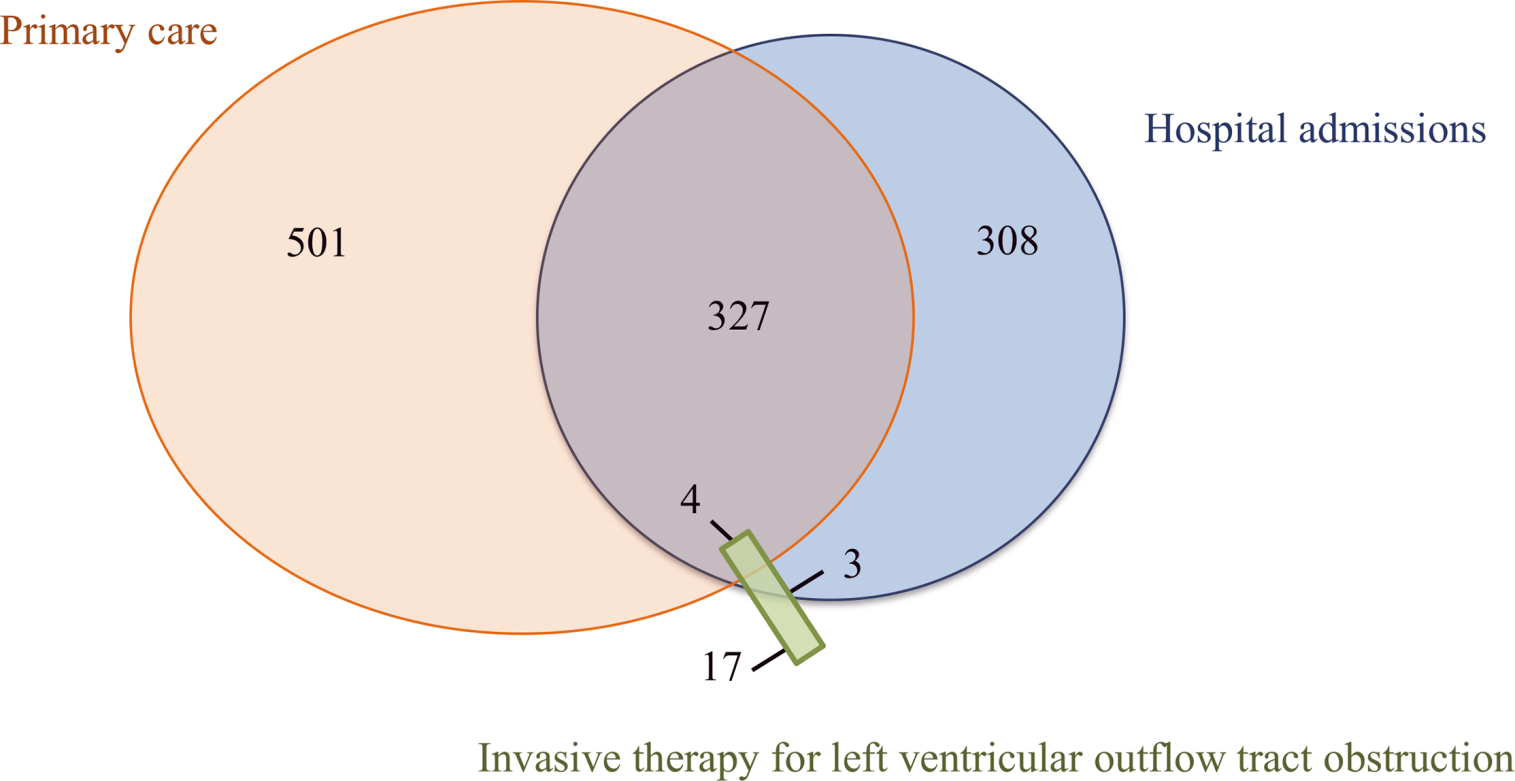
Capture of hypertrophic cardiomyopathy diagnosis in hospital admissions, primary care or both in a national sample of 5,372,790 people.

To identify unmet clinical need in the HCM population, we compared incidence rates for each endpoint using random-effects Poisson models with 95% confidence intervals (CI). Poisson models were used instead of proportional hazard Cox models because the survival curves for people with HCM and without HCM had hazard functions for several of the studied outcomes that were not proportional over time. Within practice correlation was modelled by applying random effects and allowing for clustering within practices. Models were adjusted for established cardiovascular risk factors, including sex, age (linear and quadratic linear terms), quintiles of index of multiple deprivation, smoking status, diabetes and systolic blood pressure (linear term). Missing covariate data were handled using multiple imputation by chained equations (Text A in S1 File). In secondary analyses, we tested whether associations between HCM and study endpoints differed by sex, hypertension status at entry and the geographical location of the practice, using likelihood ratio tests for interaction. We also described the causes of death in people with and without HCM. The following sensitivity analyses were performed to assess the robustness of the findings to changes in the study definitions: i) exclusion of people with diagnosed with HCM and other cardiomyopathies; ii) restriction to people with HCM who had recorded supporting information for diagnosis; iii) comparison of estimates according to the source of HCM diagnosis (exclusively in primary care vs. hospital admissions; Fig 2).

Third, to provide data relevant to trial design, we estimated the incident rates of first occurring endpoint (regardless of other endpoints) during the time of follow-up per 1000 person-years and the 3-year Kaplan-Meier cumulative estimates with 95%CIs.

Analyses were done in Stata version 13.1 (StataCorp, College Station, Texas). Statistical significance was defined as *P*<0.05.

### Ethics

Approval was granted by the Independent Scientific Advisory Committee of the Medicines and Healthcare Products Regulatory Agency and the MINAP Academic Group (Number 13_096R) and the MINAP Academic Group. The study was registered at clinicaltrials.gov (trial registration NCT02424994). Access to de-identified data for authorised researchers is provided within the UCL data safe haven. Linked CALIBER data (primary care data, Hospital Episode Statistics and Office for National Statistics mortality data) were obtained from the Clinical Practice Research Datalink (www.cprd.com). Access to de-identified data is only available once approval has been obtained through the individual constituent entities controlling access to the data (https://www.cprd.com/dataAccess/). The phenotype algorithms described in this paper are shown in the Supporting Information (Table 1 in S1 File) and are also freely available via the CALIBER website at www.caliberresearch.org and the CALIBER data portal is available for consultation online at http://www.caliberresearch.org.

## Results

### Patient characteristics

We identified 1,375 cases of HCM among the 3,3 million eligible CALIBER patients (4 per 10,000). In total 1,160 people with HCM and 11,304 individuals without HCM (10 matches for each of 864 and 9 for each of the remaining 296 individuals without HCM) were included in the analysis. Median age at first recorded diagnosis was 57 years [IQR 43–70] and 40.9% were women (Table 1). Median duration since registration in the general practice was 11.6 years. At the time of diagnosis, or within the preceding year, 17.8% received statins, 53.9% blood pressure lowering medication, 9.0% anticoagulant drugs (23.9% of patients with history of AF received warfarin) and 8.7% amiodarone. Mean systolic blood pressure was similar in people with and without HCM but higher proportions of individuals with HCM had recorded history of co-existing cardiovascular disease at the time of HCM diagnosis (47.2% vs. 2.5%). The most common cardiovascular diseases recorded before the study entry among people with HCM were stable angina (24.3%), AF (16.1%) and HF (12.8%).

**Table 1:** Characteristics of a national sample of people with and without hypertrophic cardiomyopathy.

	People with HCM (n = 1,160)	People without HCM (n = 11,304)
***Sociodemographic factors***		
Age in years, mean (SD)	55.8 (19.9)	54.9 (19.6)
Women, n (%)	476 (41.0)	4,620 (40.9)
Index of multiple deprivation in quintiles, n (%)		
1 (least deprived)	225 (19.5)	2,261 (20.1)
5 (most deprived)	248 (21.5)	2,232 (19.8)
Ethnicity, n (%)		
White	756 (91.3)	5,387 (91.8)
Asian	43 (5.2)	209 (3.6)
Afro-Caribbean	19 (2.3)	157 (2.7)
Other	10 (1.2)	113 (1.9)
Geographical location of GP practices, n (%)		
North England	231 (19.9)	2280 (20.1)
East Midlands and West England	317 (27.3)	317 (27.0)
London and South England	612 (52.8)	612 (52.9)
Duration of registration in years, median [IQR]	9.7 [2.3–18.8]	11.8 [5.4–21.3]
No. of consultations in previous year	7 [3–12]	4 [1–7]
No. of hospitalisation in previous year	0 [0–1]	0 [0–0]
***Personal and family history of cardiac disease***		
Congenital cardiomyopathy, n (%)	6 (0.5)	-
Other cardiomyopathies, n (%)	85 (7.3)	-
History of any prior CVD^a^, n (%)	548 (47.2)	294 (2.6)
***Cardiovascular risk factors***		
Smoking, n (%)		
Current	9 (12.7)	1,050 (13.0)
Former	22 (31.0)	1,961 (24.3)
Never	40 (56.3)	5,067 (62.7)
Diabetes	74 (6.4)	564 (5.0)
Hypertension	696 (60.0)	5,647 (50.0)
Systolic blood pressure in mmHg, mean (SD)	136 (21.3)	139 (18.6)
Body mass index in kg/m^2^, mean (SD)	27.3 (5.2)	27.7 (5.7)
Total cholesterol in mmol/L, mean (SD)	5.0 (1.2)	5.3 (1.1)
HDL cholesterol in mmol/L, mean (SD)	1.3 (0.4)	1.4 (0.4)
LDL cholesterol in mmol/L, mean (SD)	2.8 (1.0)	3.1 (1.0)
Triglycerides in mmol/L, mean (SD)	1.6 (0.8)	1.6 (0.9)
Serum creatinine in mg/L, mean (SD)	98.2 (32.5)	89.4 (21.1)
***Medication use in previous year***		
Any blood pressure lowering medication, n (%)	625 (53.9)	2,624 (23.2)
Beta-blockers	117 (10.1)	67 (0.6)
Calcium antagonists	115 (9.9)	66 (0.6)
ACEI/ARBs	276 (23.8)	1166 (10.3)
Amiodarone, n (%)	97 (8.4)	9 (0.1)
Statins, n (%)	206 (17.8)	747 (6.6)
Antiplatelet drug, n (%)	269 (23.2)	609 (5.4)
Aspirin, n (%)	255 (22.0)	598 (5.3)
Anticoagulant drug, n (%)	104 (9.0)	88 (0.8)

Note: HCM, hypertrophic cardiomyopathy; HDL, high-density lipoprotein; LDL, low-density lipoprotein; SD, standard deviation. Missing data (%): index of multiple deprivation 0.4%; ethnic group 46.3%; body mass index 42.8%; systolic blood pressure 19.9%; serum c-reactive protein 56.7%; smoking 28.3%; total cholesterol 64.6%; HDL cholesterol 73.4%; LDL cholesterol 77.1%; triglycerides 72.5%; serum creatinine 56.7%.

^a^History of CVD included record of previous episodes of any of the 14 cardiovascular endpoints investigated.

### Unmet clinical need: Relative incidence of fatal and non-fatal endpoints in HCM vs controls

**Fig 3:**
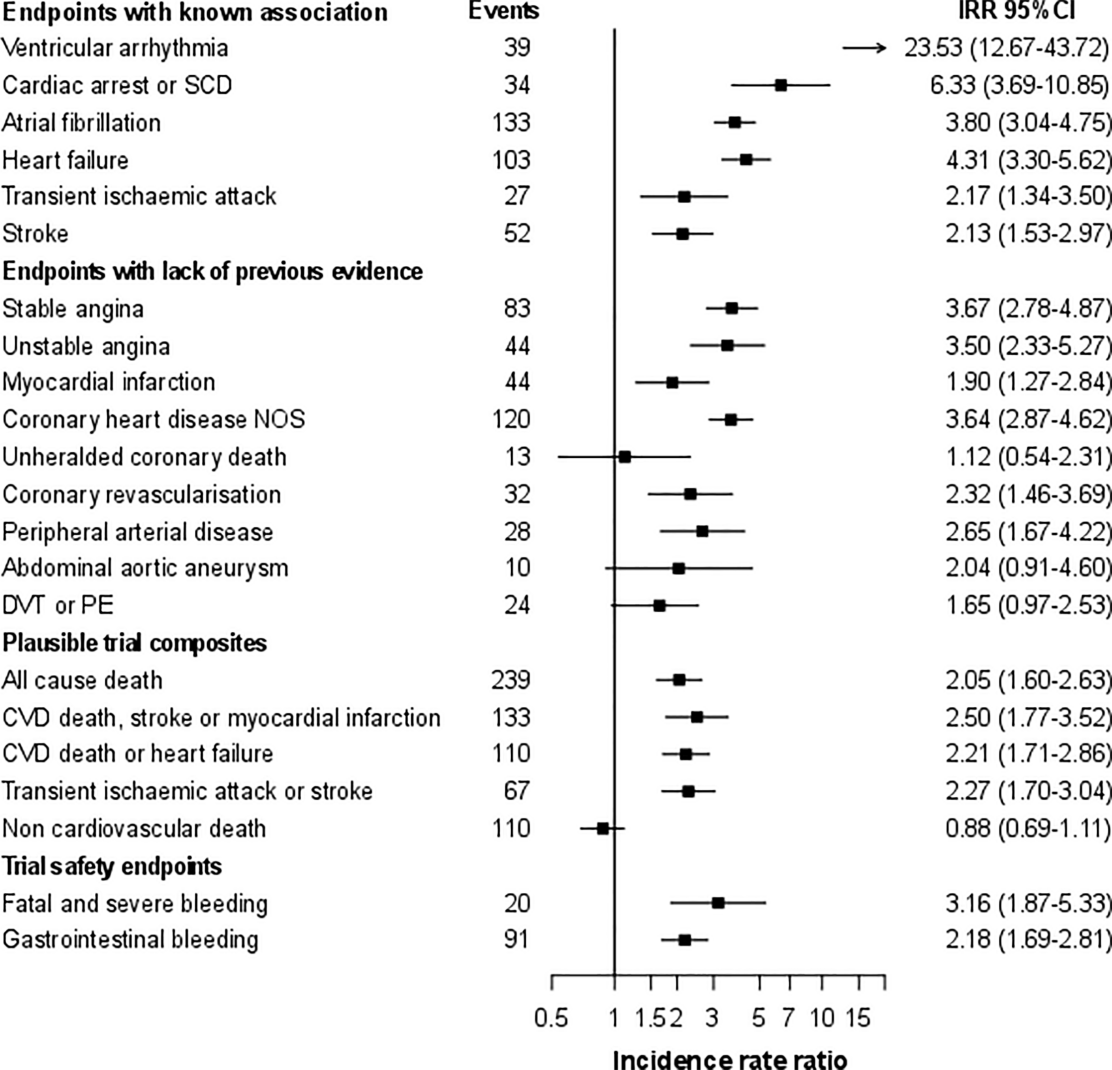
Relative risks (95% CI) of fatal and non-fatal clinically relevant and trial safety endpoints in a national sample of people with hypertrophic cardiomyopathy (vs. no hypertrophic cardiomyopathy). [Fig note: CI, confidence interval; DVT, deep vein thrombosis; MI, myocardial infarction; IRR, incidence rate ratios from random effects Poisson models adjusted for age, sex, index of multiple deprivation, smoking status, diabetes and systolic blood pressure; PE, pulmonary embolism; SCD, sudden cardiac death.].

Overall, individuals contributed 62,856.8 person-years of follow-up, median of 4.0 years. As expected, compared to people without HCM, individuals with HCM had much higher incidence of CA-SCD (adjusted IRR = 6.33, 3.69–10.85), ventricular arrhythmia (IRR = 23.53, 95%CI 12.67–43.72), AF (IRR = 3.80, 95%CI 3.04–4.75), stroke (IRR = 2.13, 95%CI 1.53–2.97) and HF (IRR = 4.31, 3.30–5.62; Fig 3). People with HCM had also increased incidence of recorded coronary and PAD and of cardiovascular composite endpoints. In particular people with HCM had higher incidence of MI (IRR = 1.90, 95%CI 1.27–2.84), coronary revascularisation (IRR = 2.32, 95%CI 1.46–3.69), and a composite of cardiovascular death, stroke or MI (IRR = 2.50, 95%CI 1.77–3.52) No evidence of interaction by sex was found except for PAD (IRR = 1.54, 95%CI 0.73–3.22 for men; IRR = 4.51, 95%CI 2.43–8.38 for women; interaction p-value = 0.03; Figure B in S1 File). Rate ratios were similar regardless of the geographical location of the general practice (Figure C in S1 File). However, higher estimates were generally observed in people without hypertension than amongst those with hypertension at baseline (Figure D in S1 File). The median age at endpoint occurrence was lower for people with HCM except for PAD and DVT-PE. Larger differences in the age at presentation compared to people without HCM were observed for cardiac arrest and ventricular arrhythmias (26 and 12 years, respectively).

### Absolute risk of plausible composite efficacy and safety endpoints

Incidence rates and 3-year Kaplan-Meier estimates for key clinical and safety endpoints relevant for trial design are shown in Table 2. Cumulative endpoint estimates among people with HCM at 3 years were 6.7% for HF, 8.8% for a composite of cardiovascular death or HF and 8.4% for a composite of cardiovascular death, stroke or MI. Estimates for trial safety endpoints were 1.5% for major bleeding and 6.0% for gastrointestinal bleeding. The most common coded clinical presentations in people with HCM but no prior history of cardiovascular endpoints were AF, HF and coronary heart disease (Figure E in S1 File).

**Table 2:** Incidence rates of endpoints and 3-year Kaplan-Meier estimates in a national sample of people with and without hypertrophic cardiomyopathy.

	People with HCM N = 1,160			People without HCM N = 11,304		
	No. of incident events	Rate / 1000 PY (95% CI)	3 year Kaplan-Meier estimates as % (95% CI)	No. of incident events	Rate / 1000 PY (95% CI)	3 year Kaplan-Meier estimates as % (95% CI)
**Endpoints with known association**						
Ventricular arrhythmia	39	8.32 (6.09–11.40)	2.27 (1.42–3.61)	18	0.31 (0.20–0.49)	0.08 (0.04–0.17)
Cardiac arrest or SCD	34	7.18 (5.13–10.05)	2.15 (1.33–3.46)	56	0.97 (0.74–1.26)	0.23 (0.15–0.35)
Atrial fibrillation	133	35.12 (29.63–41.62)	9.41 (7.41–11.92)	488	8.70 (7.96–9.50)	2.31 (2.02–2.65)
Heart failure	103	25.29 (20.85–30.68)	6.67 (5.00–8.87)	367	6.43 (5.80–7.12)	1.84 (1.58–2.15)
Transient ischaemic attack	27	5.87 (4.03–8.57)	2.03 (1.22–3.37)	155	2.70 (2.31–3.16)	0.78 (0.62–0.99)
Stroke	52	11.24 (8.56–14.75)	2.81 (1.83–4.31)	324	5.66 (5.08–6.31)	1.56 (1.32–1.84)
**Endpoints with lack of previous evidence**						
Stable angina	83	25.40 (20.48–31.50)	8.93 (6.86–11.59)	449	8.01 (7.31–8.79)	2.48 (2.17–2.83)
Unstable angina	44	9.95 (7.41–13.38)	2.61 (1.66–4.09)	158	2.75 (2.36–3.22)	0.81 (0.64–1.03)
Myocardial infarction	44	9.93 (7.39–13.34)	2.29 (1.40–3.74)	276	4.82 (4.28–5.42)	1.34 (1.12–1.61)
Coronary heart disease NOS	120	34.48 (28.83–41.24)	9.88 (7.74–12.57)	617	10.93 (10.10–11.83)	2.83 (1.50–3.20)
Unheralded coronary death	13	2.67 (1.55–4.59)	1.10 (0.54–2.20)	133	2.30 (1.94–2.72)	0.64 (0.49–0.83)
Coronary revascularisation	32	6.86 (4.85–9.70)	1.84 (1.11–3.06)	170	2.96 (2.55–3.44)	0.77 (0.61–0.98)
Peripheral arterial disease	28	5.95 (4.11–8.62)	1.50 (0.83–2.71)	147	2.56 (2.18–3.01)	0.65 (0.50–0.84)
Abdominal aortic aneurysm	10	2.07 (1.11–3.85)	0.25 (0.06–1.00)	64	1.11 (0.87–1.42)	0.24 (0.16–0.37)
DVT or PE	24	5.10 (3.42–7.61)	0.89 (0.71–1.11)	206	3.63 (3.17–4.17)	2.27 (1.42–3.61)
**Plausible trial composites**						
All cause death	239	49.12 (43.27–55.76)	14.16 (11.98–16.70)	1,307	22.59 (21.40–23.85)	5.83 (5.37–6.34)
CVD death, stroke or MI	115	26.25 (21.86–31.51)	8.38 (6.58–10.64)	496	8.70 (7.97–9.50)	2.40 (2.09–2.74)
CVD death or heart failure	110	27.06 (22.45–32.62)	8.78 (6.86–11.21)	516	9.04 (8.29–9.85)	2.46 (2.16–2.81)
Transient ischaemic attack or stroke	67	15.10 (11.89–19.19)	4.14 (2.90–5.90)	430	7.56 (6.88–8.31)	2.12 (1.83–2.44)
Non-cardiovascular death	109	22.39 (18.56–27.01)	6.63 (5.11–8.59)	859	14.84 (13.89–15.87)	3.87 (3.48–4.29)
**Trial safety endpoints**						
Major bleeding	20	4.12 (2.66–6.39)	1.47 (0.83–2.59)	92	1.59 (1.30–1.95)	0.39 (0.28–0.55)
Gastrointestinal bleeding	91	21.84 (17.79–26.83)	5.99 (4.47–7.99)	589	11.04 (10.18–11.97)	3.08 (2.72–3.48)

Note: CI, confidence interval; CVD, cardiovascular disease; DVT, deep vein thrombosis; HCM, hypertrophic cardiomyopathy; MI, myocardial infarction; PE, pulmonary embolism; SCD, sudden cardiac death. Non-disease estimates are obtained among up to 10 randomly selected patients without hypertrophic cardiomyopathy matched for sex, age, medical practice and index date; PY, person-years of follow-up.

### Unmet clinical need: HCM and cause specific mortality

A total of 1,597 deaths were recorded during follow-up, 275 (23.7%) in people with HCM and 1,322 (11.7%) in non-HCM patients. Sixty-two percent of people with a recorded cause of death (n = 1,550) died of non-cardiovascular death (44.5% and 67.5% in the HCM and non-HCM groups, respectively; Figure F in S1 File). All-cause and cardiovascular mortality rates were higher in people with HCM, with ratios of 2.05 (95%CI 1.60–2.63) for all-cause and 2.28 (95%CI 1.93–2.69) for cardiovascular death. However, no differences in mortality rates between people with and without HCM were observed for non-cardiovascular death (IRR = 0.88, 95% 0.69–1.11). Diseases of the myocardium and ischaemic heart diseases were the most common recorded causes of cardiovascular death in people with HCM (Figure G in S1 File). No evidence of difference in rates of fatal CA-SCD, HF or stroke was found between people with or without HCM. However, fatal MI was higher in people with HCM (IRR = 4.31, 95%CI 1.69–11.03). Estimates of associations between HCM and fatal endpoints did not differ by sex, hypertension status or the geographical location of the general practice.

### Sensitivity analyses

Results were robust to sensitivity analyses excluding people with HCM who had other cardiomyopathies diagnosed (n = 91), when restricted to people with HCM and supporting information of HCM (n = 690) and in people with HCM who had diagnosis exclusively recorded in primary care or in hospital (Figures H and I in S1 File).

## Discussion

We examined a national sample of population-based EHRs for 3 million individuals and made three novel findings. First, we demonstrate a methodology for using structured national EHRs in ambulatory (primary) care and hospital practice that provides the basis of a national quality and outcome registry for rare disease adaptable for use in other countries. Second, despite modern management guidelines, there is major unmet clinical need in patients with HCM illustrated by a substantial excess of preventable fatal and non-fatal endpoints including HF, AF and thromboembolism. Third, we provide accurate risk estimates of composite efficacy and safety endpoints to inform future trial design in this disease.

We chose to study HCM as–despite the fact that it is uncommon–it is a potentially fatal inheritable disease amenable to therapeutic intervention. The novelties of this study are the record-linkage cohort design, the comparison with a population-based group of people without HCM, its contemporaneity, and the investigation of fatal and non-fatal cardiovascular endpoints that have been validated in the CALIBER dataset[4,6–11]. The longitudinal design and the analysis of patient linked EHRs covering primary, hospital and mortality data allowed the identification of a matched comparison group selected from the same population. They also permitted ascertainment of HCM status regardless of disease severity and level of care management (note many patients were managed in ambulatory care and not hospitalised with their HCM).

We developed the first phenotype algorithm for HCM to identify people diagnosed with this disease using national structured EHR based on codes for diagnosis, procedures and drugs. While there are some limitations inherent in using national data for this specific disease–for example, a lack of corroborative echocardiographic imaging and genetic testing or the under-recording of information about family history of the disease (i.e. only recorded for 5% of people with HCM)–the HCM algorithm is transferrable to other countries which use SNOMED-CT and ICD-10 or similar ontologies[23]. A diagnosis of HCM is sufficiently specific that it is likely to be used only where the index of clinical suspicion is high. In addition, the very high relative and absolute risk of ventricular arrhythmia, CA-SCD and HF observed in patients with HCM is strong evidence of the diagnostic validity of the EHR algorithm. We nevertheless cannot however completely rule out the misclassification of a minority of patients wrongly labelled as diagnosed with HCM with clinical characteristics similar to HCM (e.g. patients with hypertension related enlargement of ventricles [24]). Indeed, 60% of people with HCM in our study were considered to have hypertension, which we defined as a recorded diagnosis of hypertension or a minimum of 3 high blood pressure readings recorded in the year prior to data entry. The proportion of people with HCM who had a recorded diagnosis of hypertension in our cohort was of 50%. This proportion is only slightly higher than estimates reported in previous studies (46–47% [25,26]). Unfortunately, in our data it is not possible to determine what patients were diagnosed and/or regularly treated by cardiologists.

This is the first study to determine the incidence of a wide range of fatal and non-fatal cardiovascular endpoints in people with HCM compared to random sample of individuals without the disease drawn from the general population. Contemporary outcome studies from specialist referral centres suggest that the prognosis of managed cohorts is relatively benign in terms of life expectancy[27,28]. However, this analysis of national EHRs suggests that there is, instead, a substantial unmet need with respect to the risk of complications that associates with a poorer long-term survival compared to the normal population. The risk of some of these complications–for example, sudden cardiac death and stroke–may be lowered if existing practice guidelines are implemented. We provide evidence that current clinical guidelines for HCM are not being followed. For example, more than a quarter of patients with HCM and AF were not treated with anticoagulants in spite of clear recommendations in practice guidelines to do so. Similarly, the high incidence of CA-SCD suggests that risk stratification and prophylactic implantable cardioverter defibrillators are underutilised.

Unexpectedly, we found that people with HCM were at higher risk of MI and coronary revascularisation compared to population controls. This novel finding is difficult to explain with our current understanding of the pathophysiology of HCM. It might result from misdiagnosis in patients with elevated serum troponin levels in the presence of angiographically normal coronary arteries[29], but this does not explain the small but significant coronary revascularisation excess. People with HCM were also more likely to be prescribed statins and antiplatelets than population controls; but again this may be explained not by an excess of atherosclerotic disease but rather by the indiscriminate use of secondary prevention in symptomatic people with HCM. This latter point is not trivial as patients with HCM may be exposed unnecessarily to polypharmacy and side-effects such as bleeding.

With respect to the second aim of this study, future trial design, we provide data that can be used to estimate sample size and the length of treatment required to power randomised interventions targeting specific endpoints such as stroke, progressive HF and ventricular arrhythmia. For the first time, we examine a specific safety endpoint–bleeding risk–in comparison to a normal matched population. This will be of particular interest in trials designed to reduce the high rate of thromboembolism seen in this study. Finally, these same primary care data are a potential route back to patients, in that they can be contacted for randomization via CPRD.

More broadly this study has implications for the approach to other uncommon and rare diseases. Deep, computable efforts at characterizing and annotating phenotype algorithms in rare diseases have used the human phenotype ontology (HPO). It is possible to map 92% of the 10,454 HPO concepts to SNOMED CT (30% complete mapping and 62% partial) suggesting the potential of power of national EHR to identify and perform unbiased analysis of much rarer conditions that are otherwise very difficult to study[23]. For example, the CPRD, which provided the primary care data in this study, also provides services to randomise patients at the point of care[30].

Potential limitations not already discussed include the possibility that temporal or provider changes in clinical diagnosis and management of patients might affect differences in incidence of endpoints between HCM and non-HCM patients. However, the matching design for practice and date of study inclusion minimised the likelihood of this type of bias. We were also unable to distinguish between people with or without left ventricular hypertrophy and the large number of missing baseline lipid values prevented adjustment of ratios for this cardiovascular risk factor.

In summary, this study identified major unmet clinical need in HCM and highlights the importance of implementing improved cardiovascular prevention strategies that increase the life-expectancy of people with HCM. It also provided clinically meaningful risks of safety and composite efficacy endpoints essential for trial design, demonstrating the value of national EHR in bringing treatment trials to rare and uncommon diseases.

## Supporting information

S1 File Supplemental methods. (DOC)
