# Supplementary material for: Identifying unmet clinical need in hypertrophic cardiomyopathy using national electronic health records
Source: PLoS One. 2018 Jan 11;13(1):e0191214. doi: 10.1371/journal.pone.0191214 (PMC5764451; doi:10.1371/journal.pone.0191214)
Supplement: S1 File — (DOC) [file pone.0191214.s001.doc]

**Supplemental methods**

**Text A. Data sources and multiple imputation**

**Data sources**

Four sources of data were linked for this study by the Clinical Practice Research Datalink (CPRD; [**www.cprd.com**](http://www.cprd.com/)): the CPRD, the Hospital Episodes Statistics (HES; [**http://www.hscic.gov.uk/hes**](http://www.hscic.gov.uk/hes)), the Myocardial Infarction National Audit Project (MINAP; [**https://www.ucl.ac.uk/nicor/audits/minap**](https://www.ucl.ac.uk/nicor/audits/minap)), and cause-specific mortality from the Office of National Statistics (ONS; [**http://www.ons.gov.uk/ons**](http://www.ons.gov.uk/ons)). CPRD provides all health related information recorded in primary care, including demographic and health behaviour data, clinical diagnoses, symptoms, clinical and laboratory tests, medical procedures and prescriptions. CPRD data are coded with the Read clinical code system. The primary care practices in CPRD and the subset included in the CALIBER platform, are representative of the UK primary care setting[^1^](#_ENREF_1) and have been validated for epidemiological research[^2-9^](#_ENREF_2). HES provides information about diagnoses and medical procedures during all elective and emergency hospital admission across all National Health Service hospitals in England. Both HES and ONS data linked in CALIBER are coded with the 10th revision of the International Classification of Diseases (ICD-10). ONS data is coded with the 9^th^ revision of the ICD before the year 2000. HES also includes the version 4^th^ of the Office of Population Censuses and Surveys Classification of Intervention and Procedures (OPCS-4) for all intra-hospital medical and surgical procedures. MINAP is a national registry of patients with acute coronary syndromes admitted to hospitals across the UK.

Phenotyping algorithms combining Read, ICD-9/10, OPCS-4 to define covariates and endpoints are available online through CALIBER portal ([**https://www.caliberresearch.org/portal**](https://www.caliberresearch.org/portal)).

**Multiple imputation**

Risk factor data appeared to be missing at random after adjusting for major confounders (e.g. age, sex, diabetes, BMI and blood pressure). Hence, multiple imputation was implemented using the *‘mi’*[*^10^*](#_ENREF_10) algorithm in the Stata software version 13.1 (StataCorp LP, College Station, USA), to replace missing values in risk factor variables. Imputation models included:

1. all the baseline covariates used in the main analysis (sex, age, quadratic age, index of multiple deprivation, diabetes, smoking, body mass index and systolic blood pressure);
2. prior (between 1 and 4 years before study entry) and post (between 0 and 1 year after study entry) averages of continuous covariates in the main analysis;
3. baseline, prior and post average measurements of covariates not considered in the main analysis (diastolic blood pressure, pulse rate, total cholesterol, HDL cholesterol, LDL cholesterol, creatinine);
4. year of study entry, number of consultations in the year prior to study entry, number of hospitalisations in the year prior to study entry;
5. baseline medications (antiplatelet medication, statins, other lipid lowering medication, blood pressure lowering medication, low-dose aspirin, anticoagulant medication);
6. coexisting medical conditions (history of cardiovascular disease, cancer, renal disease, liver disease and chronic obstructive pulmonary disease);
7. the Nelson-Aalen hazard, incident cardiovascular disease status for each of the endpoints analysed (binary variable)^2^, death and time of death since study entry.

Non-normally distributed variables were log-transformed for imputation and back-exponentiated to their original scale for analysis. Plausibility of imputations for all covariates was checked by comparing plots of the distribution of recorded and imputed values[^11^](#_ENREF_11). Five multiply imputed datasets were generated, and Poisson models were fitted to each dataset. Coefficients were combined using Rubin’s rules. The Kolmogorov-Smirnov test was used to compare the distribution of observed versus imputed log-transformed covariates.

**References**

1. Walley T, Mantgani A. The UK General Practice Research Database. *Lancet.* Oct 11 1997;350(9084):1097-1099.

2. George J, Rapsomaniki E, Pujades-Rodriguez M, et al. How Does Cardiovascular Disease First Present in Women and Men? Incidence of 12 Cardiovascular Diseases in a Contemporary Cohort of 1,937,360 People. *Circulation.* Sep 1 2015.

3. Herrett E, Gallagher AM, Bhaskaran K, et al. Data Resource Profile: Clinical Practice Research Datalink (CPRD). *International journal of epidemiology.* Jun 2015;44(3):827-836.

4. Pasea L, Chung SC, Pujades-Rodriguez M, et al. Personalising the decision for prolonged dual antiplatelet therapy: development, validation and potential impact of prognostic models for cardiovascular events and bleeding in myocardial infarction survivors. *European heart journal.* Feb 27 2017.

5. Pujades-Rodriguez M, Duyx B, Thomas SL, et al. Rheumatoid Arthritis and Incidence of Twelve Initial Presentations of Cardiovascular Disease: A Population Record-Linkage Cohort Study in England. *PloS one.* 2016;11(3):e0151245.

6. Pujades-Rodriguez M, George J, Shah AD, et al. Heterogeneous associations between smoking and a wide range of initial presentations of cardiovascular disease in 1937360 people in England: lifetime risks and implications for risk prediction. *International journal of epidemiology.* Feb 2015;44(1):129-141.

7. Pujades-Rodriguez M, Timmis A, Stogiannis D, et al. Socioeconomic deprivation and the incidence of 12 cardiovascular diseases in 1.9 million women and men: implications for risk prediction and prevention. *PloS one.* 2014;9(8):e104671.

8. Rapsomaniki E, Timmis A, George J, et al. Blood pressure and incidence of twelve cardiovascular diseases: lifetime risks, healthy life-years lost, and age-specific associations in 1.25 million people. *Lancet.* May 31 2014;383(9932):1899-1911.

9. Shah AD, Langenberg C, Rapsomaniki E, et al. Type 2 diabetes and incidence of cardiovascular diseases: a cohort study in 1.9 million people. *The lancet. Diabetes & endocrinology.* Feb 2015;3(2):105-113.

10. Royston P, White IR. Multiple imputation by chained equations (MICE): Implementations in STATA. *Journal of Statistical Software.* 2011;45(4):1-20.

11. van Buuren S. Multiple imputation of discrete and continuous data by fully conditional specification. *Statistical methods in medical research.* Jun 2007;16(3):219-242.

**Supplemental tables**

**Table A**. CALIBER cardiovascular endpoint definitions by data source

| **Endpoint** | **CPRD – Read codes** | **MINAP registry** | **HES-OPCS 4 procedures** | **HES-ICD 10 hospital diagnoses**† | **ONS-ICD 10 causes of death**‡ |
| --- | --- | --- | --- | --- | --- |
| **Stable angina** | G33z400, G33..00, G33z.00 + 19 other Read codes for diagnosis of stable angina pectoris.  Test results coded associated with 33 Read codes for coronary angiography, or 139 Read codes for myocardial ischaemia tests (resting ECG, exercise ECG, stress echo, radioisotope scan).  Two or more successive prescriptions for anti-anginal drugs | Not used | Not used | I20.1; I20.8; I20.9 | Not used |
| **Unstable angina** | G311500, G311100 + 10 other Read codes for unstable angina | Discharge diagnosis of acute coronary syndrome without raised troponin | Lack of coronary artery bypass graft or percutaneous coronary intervention record in the same hospital spell as I20.9 | I20.0, I24.0, I24.8, I24.9, I20.9 without coronary artery bypass graft or percutaneous coronary intervention in the same hospital spell | Not used |
| **Non-fatal acute Myocardial Infarction (MI)** | G30X000, G307100, G30..15 + 53 other Read codes  7929100: Percutaneous transluminal coronary thrombolysis with streptokinase + 3 other Read codes for coronary thrombolysis.  Elevated cardiac markers, troponin or CKMB results associated with 16 Read codes | MI with or without ST elevation based on initial ECG findings, raised troponins and clinical diagnosis | K50.2, K50.3 | I21, I22 | Not used |
| **Unheralded coronary death** | Any CVD excluded | Any CVD excluded | Any CVD excluded. | Any CVD excluded | I20-I25 |
| **Coronary revascularisation** | 57 Read codes for coronary artery bypass graft.  28 Read codes for percutaneous coronary intervention | Not used | K40-K46, K49, K50 and K75 | Not used | Not used |
| **Heart failure** | G58..00 + 40 other Read codes for heart failure, 585f.00, 585g.00 | Not used | Not used | I11.0, I13.0, I13.2, I50 | I11.0, I13.0, I13.2, I50 |
| **Ventricular arrhythmia** | G757.00 + 7 other Read codes for ventricular fibrillation | Not used. | Not used | I47.0, I47.2, I49.0 | I47.0, I47.2, I49.0 |
| **Cardiac arrest** | G575.00 + 22 other Read codes for cardiac arrest, asystole, external cardiac resuscitation | Not used | X50 | I46.0, I46.9 | I46.0, I46.9 |
| **Sudden cardiac death** | G575100 | Not used | Not used | I46,1R96 | I46, R96 |
| **Atrial fibrillation** | G573000 + 7 other Read codes for paroxysmal atrial fibrillation, persistent atrial fibrillation and atrial flutter. | Not used | Not used | I48.0 | Not used |
| **Endpoint (CONTIN.)** | **CPRD – Read codes** | **MINAP registry** | **HES-OPCS 4 procedures** | **HES-ICD 10 hospital diagnoses**† | **ONS-ICD 10 causes of death**‡ |
| **Ischaemic stroke** | G64..11 + 9 other Read codes, 7A20311 + 4 other Read codes for carotid endarterectomy within 90 days of stroke not otherwise specified | Not used | L29.5 + 3 other codes for carotid endarterectomy or stenting within 90 days of stroke not otherwise specified | I63 | I63 |
| **Subarachnoid haemorrhage** | G60X.00 + 2 other Read codes for subarachnoid haemorrhage. | Not used | Not used | I60 | I60 |
| **Intracerebral haemorrhage** | G61..00 + 16 other Read codes for intracerebral haemorrhage | Not used | Not used | I61 | I61 |
| **Unclassified stroke** | G66..00 + 14 other Read codes | Not used | U54.3 | I64, G46.3-G46.7 | I64, I67.2, I67.9 |
| **Transient ischaemic attack** | G65..12 + 5 other Read codes | Not used | Not used | G45.8-G45.9 | Not used |
| **Abdominal aortic aneurysm** | G714.00 + 12 other Read codes. | Not used | L18-20, L254, L27, L28 | I71.3, I71.4, I71.5, I71.6, I71.8, I71.9 | I71.3, I71.4, I71.5, I71.6, I71.8, I71.9 |
| **Peripheral arterial disease (PAD)** | 63 codes for Lower limb peripheral arterial disease diagnosis, 136 Read codes for PAD procedures, 2 Read codes for abnormal lower limb angiogram. | Not used | L50-L54, L58-L60, L62, L65 | I73.1, I73.8, I73.9, I74.3, I74.4, I74.5 | I73.1, I73.8, I73.9, I74.3  I74.4, I74.5 |
| **Deep vein thrombosis** | G801.00 + 8 other Read codes | Not used | Not used | I80.1, I80.2, I80.3 | I80.1, I80.2, I80.3 |
| **Pulmonary embolism** | G401.00 + 14 other Read codes for pulmonary embolism + 3 Read codes for pulmonary embolectomy. | Not used | Not used | I26 | I26 |
| **Bleeding** | 7L14311 + 9 other Read codes for transfusion within 30 days of a hospital admission for any of the bleeding codes listed in the ONS source | Not used | X33 + 5 other OPCS codes for transfusion within 30 days of a hospital admission for any of the bleeding codes listed in the ONS source | I60, I61, I62, or  primary hospitalisation with a hospital duration >14 days or a death recorded within 7 days for any of the bleeding codes listed in the ONS source | I60, I61, I62, K25 + 20 other ICD10 codes for gastrointestinal ulcer or bleeding, P26.1 + 4 other ICD10 codes for bleeding of respiratory sites, H35.6 + 2 other ICD10 codes for ocular bleeding |

**Table B**. Baseline patient characteristics of people with hypertrophic cardiomyopathy who had or not recorded supporting information for diagnosis

|  | HCM individuals with recorded supporting data  (n=690) | HCM individuals without recorded supporting data  (n=470) |
| --- | --- | --- |
| ***Sociodemographic factors*** |  |  |
| Age in years, mean (SD) | 58.4 (18.0) | 51.9 (21.7) |
| Women, n (%) | 291 (42.2) | 185 (39.4) |
| Index of multiple deprivation in quintiles, n (%) |  |  |
| 1 (least deprived) | 124 (18.0) | 101 (21.6) |
| 5 (most deprived) | 147 (21.4) | 101 (21.6) |
| Duration of registration in years, median [IQR] | 10.0 [3.3-19.3] | 9.2 [1.3-18.3] |
| Consultation rate in previous year | 8 [4-13] | 6 [2-11] |
| Hospitalisation rate in previous year | 0 [0-1] | 0 [0-1] |
| ***History of cardiac disease*** |  |  |
| Other cardiomyopathies, n (%) | 60 (8.7) | 25 (5.3) |
| History of any prior CVD, n (%) | 379 (54.9) | 168 (35.7) |
| ***Cardiovascular risk factors*** |  |  |
| Smoking, n (%) |  |  |
| Current | 77 (14.7) | 50 (15.3) |
| Former | 151 (28.7) | 73 (22.3) |
| Never | 298 (56.7) | 204 (52.4) |
| Diabetes | 48 (7.0) | 26 (5.5) |
| Hypertension diagnosis | 447 (64.8) | 249 (53.0) |
| Systolic blood pressure in mmHg, mean (SD) | 139 (18.6) | 136 (21.1) |
| Body mass index in kg/m^2^, mean (SD) | 27.7 (5.7) | 26.8 (5.2) |
| ***Medication use in previous year*** |  |  |
| Blood pressure lowering medication, n (%) | 427 (61.9) | 198 (42.1) |
| Statins, n (%) | 148 (21.5) | 58 (12.3) |
| Antiplatelet drug, n (%) | 188 (27.3) | 81 (17.2) |
| Aspirin, n (%) | 175 (25.4) | 80 (17.0) |
| Anticoagulant drug, n (%) | 90 (13.0) | 14 (3.0) |

Note: CVD, cardiovascular disease; HCM, hypertrophic cardiomyopathy; IQR, interquartile range; SD, standard deviation.

**Supplemental figures**

**Figure A. Study flow diagram**

5 372 790 CALIBER people

3 290 455 people eligible

1375 people with HCM

215 people without follow-up during the study period^a^

1160 people with HCM

Note: HCM, hypertrophic cardiomyopathy.

^a^The group of people who did not have follow-up during the study period left the general practice or died before 1^st^ January 1997 and/or had less than one year of follow-up since registration in the practice.

**Figure B. Associations of hypertrophic cardiomyopathy (vs. non hypertrophic cardiomyopathy) with fatal and non-fatal endpoints in men and in women**

**
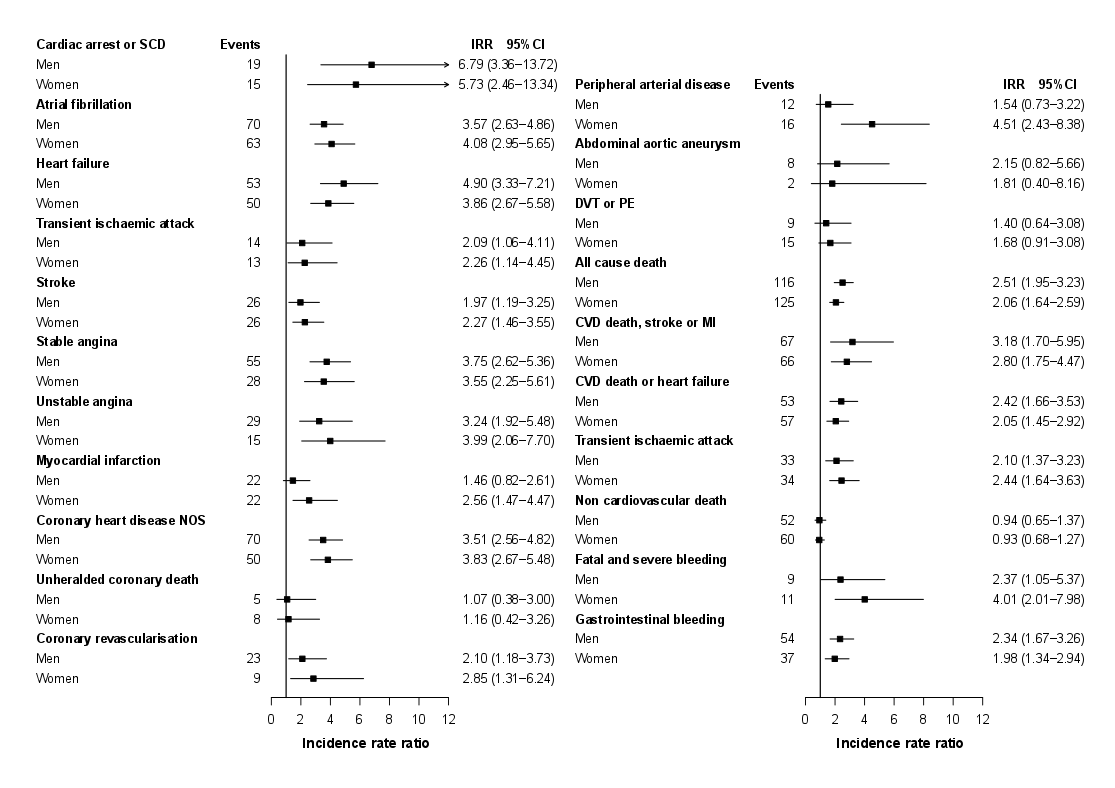
**

Note: DVT, deep vein thrombosis; IRR, incidence rate ratios from random effects Poisson models adjusted for age, sex, index of multiple deprivation, smoking status, diabetes and systolic blood pressure; MI, myocardial infarction; NOS, not otherwise specified PE; pulmonary embolism. Likelihood ratio for interaction p-value for peripheral arterial disease was 0.03 and ≥0.05 for other endpoints.

**Figure C. Associations of hypertrophic cardiomyopathy (vs. non hypertrophic cardiomyopathy) with fatal and non-fatal endpoints according to the geographical location of the general practice**

**
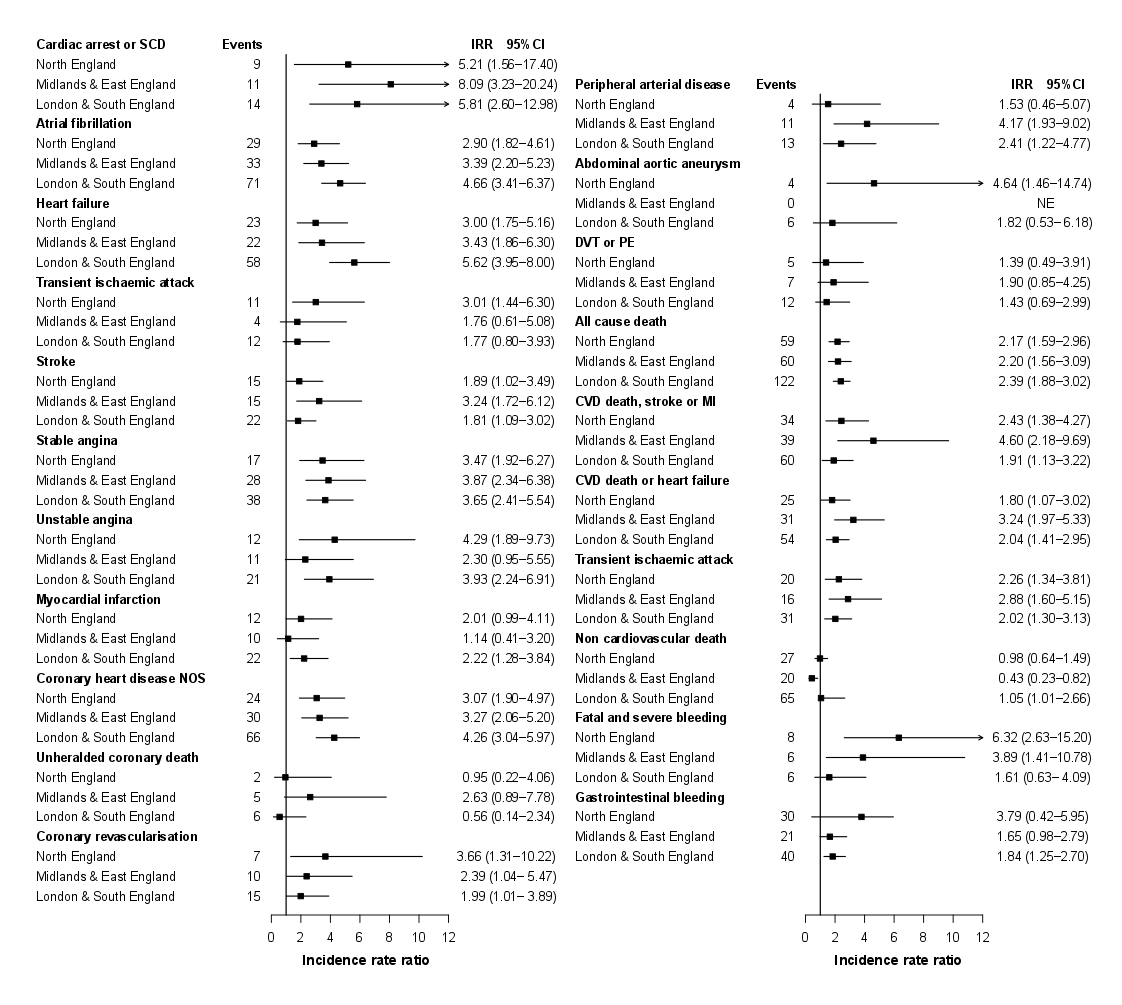
**

Note: CI, confidence interval; DVT, deep vein thrombosis; IRR, incidence rate ratios from random effects Poisson models adjusted for age, sex, index of multiple deprivation, smoking status, diabetes and systolic blood pressure; NE, not estimated because no events were recorded; NOS, not otherwise specified; PE, pulmonary embolism. A total of 231 (19.9% of HCM patients), 317 (27.3% of HCM patients) and 612 (52.8% of HCM patients) patients were registered in CPRD general practices located in North England; East Midlands and West England; and London & South England, respectively. P-value from likelihood ratio test for interaction was 0.02 for gastrointestinal bleeding and ≥0.05 for other endpoints.

**Figure D. Associations of hypertrophic cardiomyopathy (vs. non hypertrophic cardiomyopathy) with fatal and non-fatal endpoints amongst people with and without diagnosed hypertension at baseline**

**
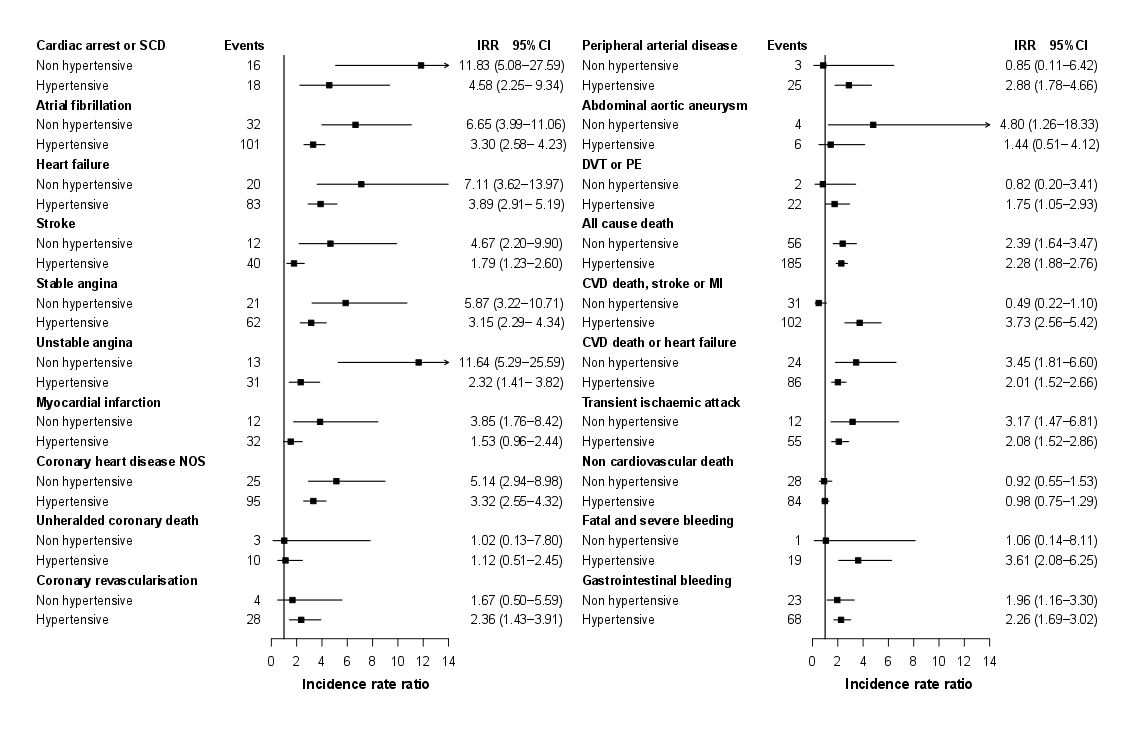
**

Note: DVT-PE, composite of deep vein thrombosis and pulmonary embolism; IRR, incidence rate ratios from random effects Poisson models adjusted for age, sex, index of multiple deprivation, smoking status, diabetes and systolic blood pressure; MI, myocardial infarction; NE, not estimated because of small number of events; NOS, not otherwise specified. P-values from likelihood ratio test for interaction were ≤0.05 for: unstable angina (0.0007), myocardial infarction (0.05), atrial fibrillation (0.02), stroke (0.03); and the composite of cardiovascular death, stroke and myocardial infarction (<0.0001). No estimates are reported for transient ischaemic attack because no events were recorded amongst people with hypertension without hypertrophic cardiomyopathy.

**Figure E. Distribution of the initial presentation of cardiovascular diseases in people with and without hypertrophic cardiomyopathy**


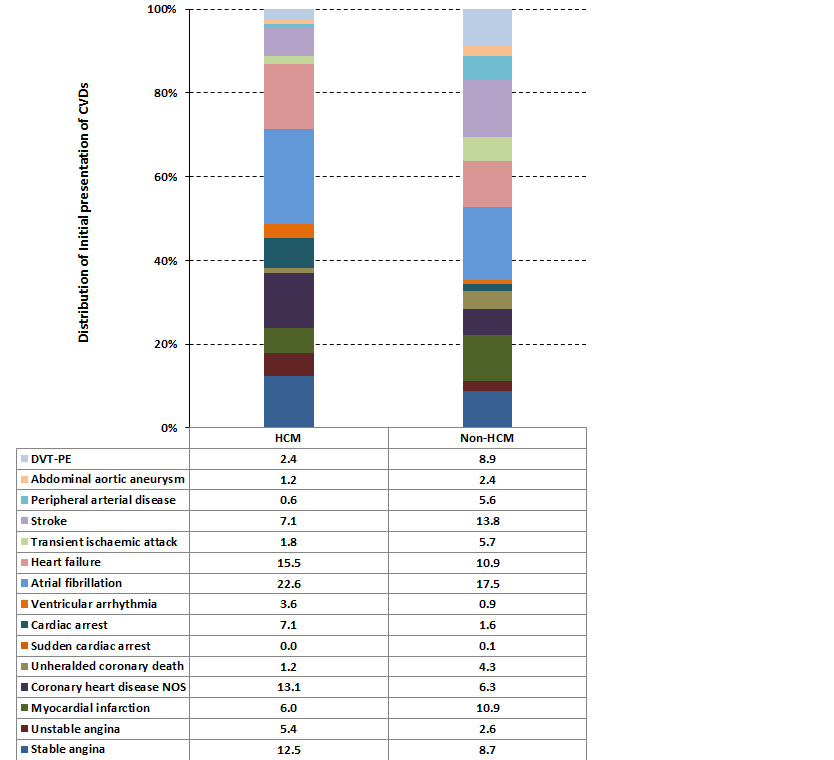


Note: DVT-PE, composite of deep vein thrombosis and pulmonary embolism; NOS, not otherwise specified. A total of 767 HCM patients and 3,329 non-HCM patients experienced an incident episode of cardiovascular disease during the study follow-up. This table shows the proportion of types of clinical presentations of cardiovascular disease.

**Figure F. Distribution of causes of death in people with and without hypertrophic cardiomyopathy**

**
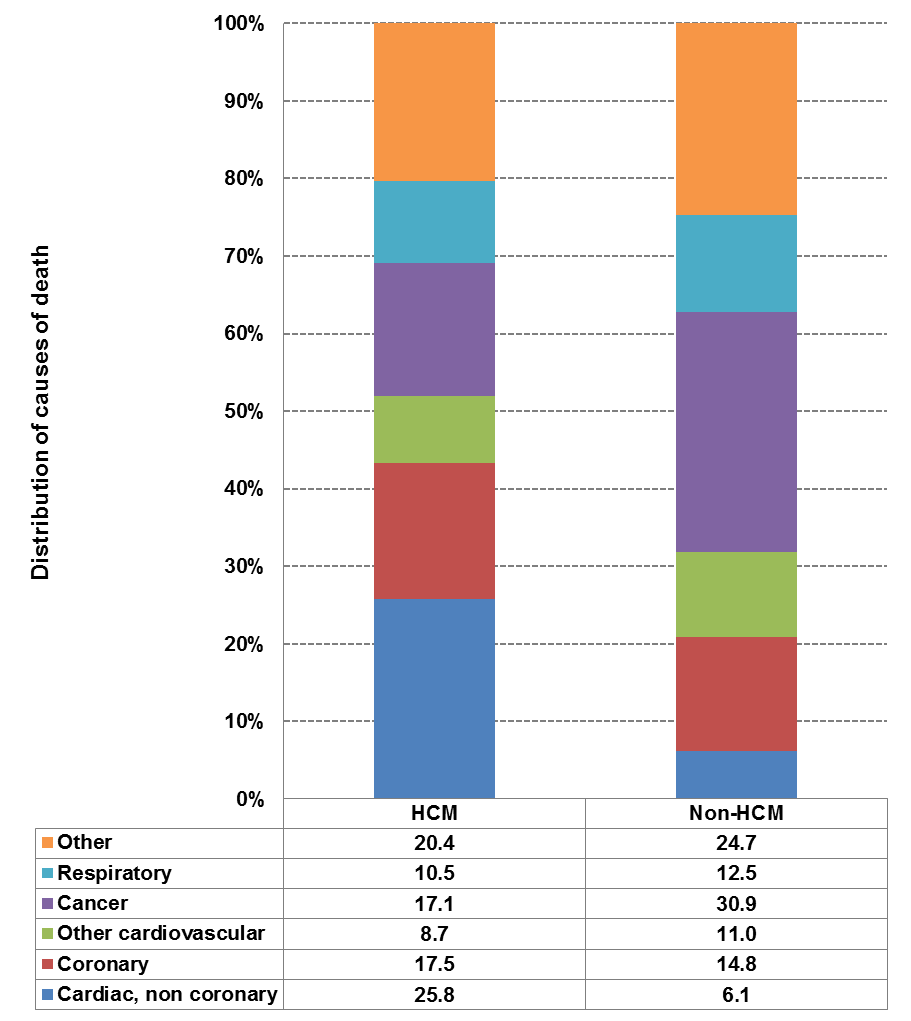
**

Note: Table shows the proportion of deaths for specific causes

**Figure G. Distribution of cardiovascular causes of death in people with and without hypertrophic cardiomyopathy**


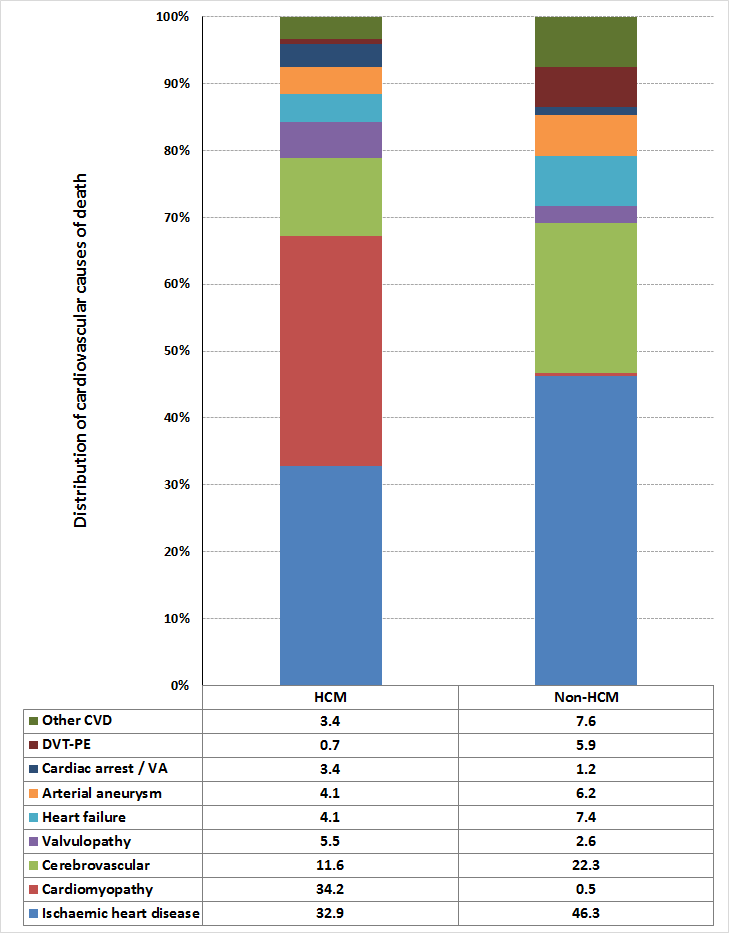


Note: CVD, cardiovascular disease; DVT-PE, composite of deep vein thrombosis and pulmonary embolism; VA, ventricular arrhythmia. Table shows the proportion of deaths for specific causes.

**Figure H. Associations of hypertrophic cardiomyopathy (vs. non hypertrophic cardiomyopathy) with fatal and non-fatal endpoints amongst people who had or not recorded other cardiomyopathies or supporting information for HCM**

**
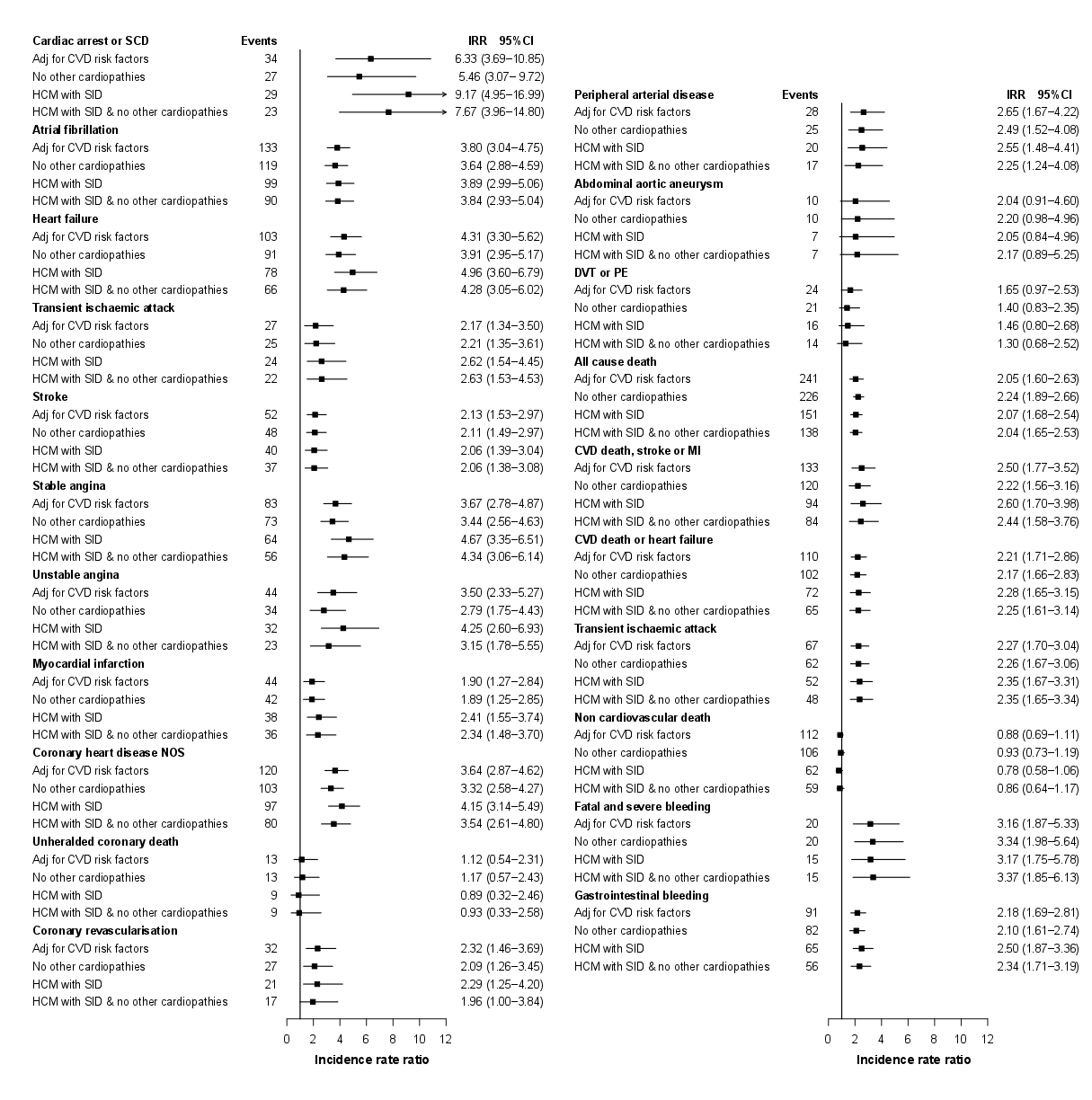
**

Note: CI, confidence interval; CVD, cardiovascular disease; DVT, deep vein thrombosis; HCM, hypertrophic cardiomyopathy; IRR, incidence rate ratios from random effects Poisson models adjusted for age, sex, index of multiple deprivation, smoking status, diabetes and systolic blood pressure; MI, myocardial infarction; NOS, not otherwise specified; PE, pulmonary embolism; SID, supporting information for HCM diagnosis. A total of 91 patients with HCM had cardiomyopathy diagnosed. A total of 690 (59.5% of HCM patients) and 6,784 (60.0% of non-HCM patients) had recorded supporting information for HCM.

**Figure I. Associations of hypertrophic cardiomyopathy (vs. non hypertrophic cardiomyopathy) with fatal and non-fatal endpoints according to the source of hypertrophic cardiomyopathy diagnosis**

**
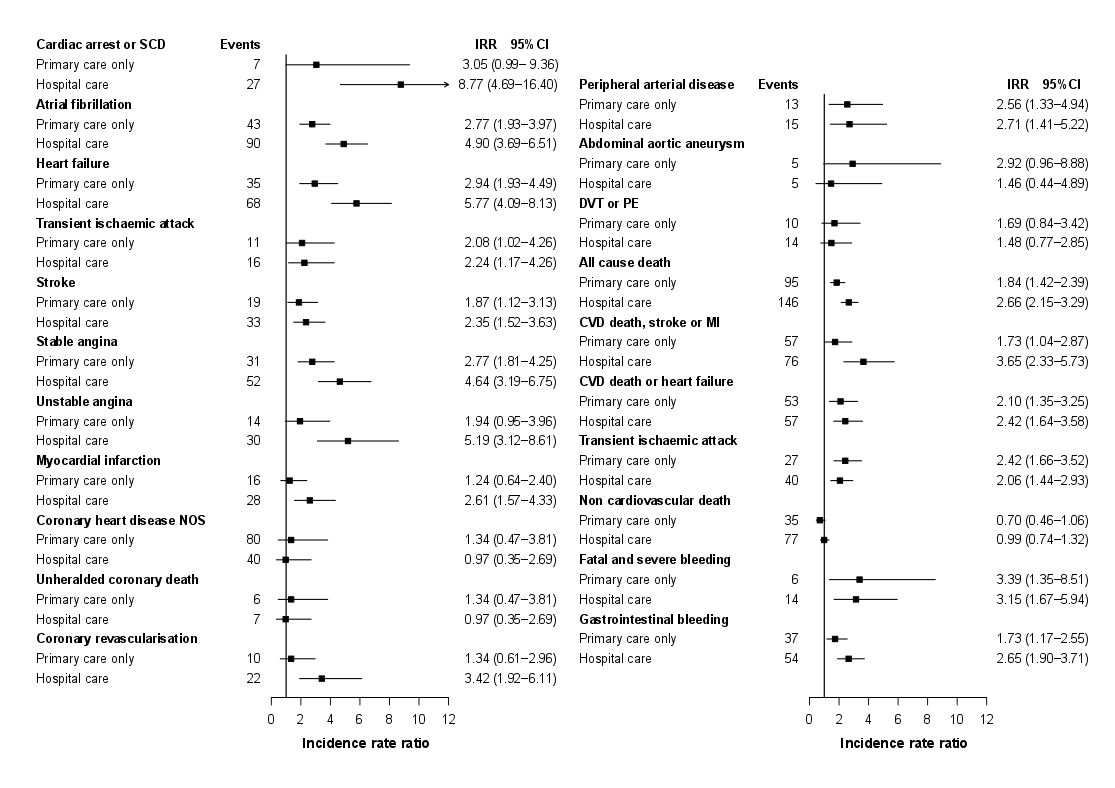
**

Note: CI, confidence interval; DVT, deep vein thrombosis; HCM, hypertrophic cardiomyopathy; IRR, incidence rate ratios from random effects Poisson models adjusted for age, sex, index of multiple deprivation, smoking status, diabetes and systolic blood pressure; NOS, not otherwise specified; PE, pulmonary embolism. A total of 501 patients with HCM were diagnosed in primary care only and 642 during hospitalisation. P-values from likelihood ratio test for interaction were ≤0.05 for: unstable angina (0.03), coronary heart disease NOS (0.01), heart failure (0.02), cardiac arrest or SCD (0.005), atrial fibrillation (0.01) and the composite of cardiovascular death, stroke and MI (0.03).
